# Rise in the Number of Complex Skin Cancers Necessitates Establishing Mohs Micrographic Surgery Fellowships in Pakistan

**DOI:** 10.7759/cureus.43183

**Published:** 2023-08-09

**Authors:** Shahan Tariq, Afshan Saeed, Hamza Ismaeel, Sana Ullah, Muhammad Ammar Hamid

**Affiliations:** 1 Dermatology, National University of Medical Sciences, Rawalpindi, PAK; 2 Dermatology, Pak-Emirates Military Hospital, Rawalpindi, PAK; 3 Dermatology, District Headquarters (DHQ) Hospital Haripur, Haripur, PAK; 4 Pediatrics, Rawalpindi Medical University, Rawalpindi, PAK; 5 Oncology, NORI Hospital, Islamabad, PAK; 6 Internal Medicine, University of South Dakota Sanford School of Medicine, Sioux Falls, USA

**Keywords:** dermato-oncology, facial plastic, reconstructive microsurgery, mohs reconstruction, fellowship of dermatology, moh's micrographic surgery, mohs surgery

## Abstract

As more and more patients seek treatment for increasingly complicated and cosmetically challenging skin cancers, Mohs Micrographic Surgery (MMS) is now exceedingly in demand. Training in MMS could help dermatologists improve patient outcomes allowing them to handle complex lesions safely and efficiently and hence, provide the best possible care. As a result, there is an urgent need to train additional dermatologists in Mohs Surgery in order to meet the huge demand for dermatologists with imperative expertise, specifically in this field.

## Editorial

Dermatological surgery has come a long way since its inception in the early 20th century. With advances in technology and surgical techniques, dermatosurgery has become a highly specialized field of dermatology. Two popular procedures in dermatosurgery are simple dermatosurgery, also referred to as wide or standard excision (SE), and Mohs Micrographic Surgery (MMS). While both procedures are used to treat skin cancer, they differ significantly in their procedures, techniques, benefits, expertise required, costs, and outcomes/prognosis.

SE is a surgical technique that involves excision of the cancerous tissue and stitching the wound back together. The procedure is performed under local anesthesia and typically takes less than an hour to complete. Prior to the procedure, the dermatologist marks the area around the cancerous tissue with a surgical pen to guide the incision. Once the tissue is removed, the wound is closed with sutures or staples. The removed tissue is then sent to a laboratory for examination to ensure that all cancerous cells have been removed. Although it is a simpler technique, SE has been associated with significantly higher rates of local persistence. Moreover, the development and refinement of newer methods of dermatological surgery, such as MMS, has led to its decline further [1].

For MMS, the tissue is removed from the cancerous area in layers and examined under a microscope in real-time until no more abnormal cells can be seen thereby, conserving a significantly larger portion of healthy tissue. Although the process is quite complex in and of itself, it generally involves a dermatologist removing thin layers of the tissue that needs to be excised and examining it under a microscope. If evidence of cancerous tissue is found, subsequent layers are removed and examined; this process continues until no more suspicious cells can be observed on the slide. Once the cancerous tissue has been completely removed, the wound is closed with sutures or left to heal on its own. This technique offers much more precise control of the complete tumor margin along with the fact that there exist no absolute contraindications to MMS in those deemed suitable for surgery in general [2].

One of multiple systemic reviews and meta-analyses conducted revealed that in comparison to SE, MMS was found to have significantly decreased incidence of recurrence and size of defect in both primary and secondary Basal Cell Carcinomas (BCC) [3]. MMS has been proven to be the ideal choice when the cancerous tissue involves complex areas such as the H-zone of the face as well as regions where the skin is of premium value with regards to cosmetics and functionality. Nevertheless, SE may sometimes be used for the management of lesions in other, not-so-complex and cosmetically non-premium, areas for several reasons [4]. In terms of outcomes/prognosis, both procedures have high cure rates, but MMS may result in a higher cure rate for certain types of skin cancer, such as BCCs, squamous cell carcinomas (SCC), melanomas, etc. The decision to choose one technique over the other and optimize procedures according to the patient is where the appropriate use criteria come into effect, ultimately bringing together the use of evidence-based medicine, and clinical experience with expert judgment [5].

Some studies have argued MMS to be costly and more time-consuming in the past; however, recent literature highlights MMS to be the superior method of surgical excision having a higher intrinsic value along with being cost-effective in the long run compared to SE. This shall only improve due to the development of more modern and refined techniques as well as the training of a larger number of dermatologists. However, it is important to note that Mohs Surgery requires specialized training and expertise. For example, in the USA, according to the American Board of Dermatology and the American College of Mohs Surgery, dermatologists may pursue a one-year dedicated fellowship in Mohs surgery [6], which incorporates knowledge of clinical and pathologic diagnoses, tumor staging specific to dermatological oncology, and treatment choices for patients with cutaneous malignancies not to mention, extensive training in surgical techniques, pathology, and reconstruction.

Currently, there are a total of 58 Fellowship of the College of Physicians & Surgeons (FCPS) and 39 Membership of the College of Physicians & Surgeons (MCPS) dermatology residency programs in the country accredited by the College of Physicians and Surgeons Pakistan (CPSP) [7,8]. However, dermatology sub-specialty fellowship programs, especially MMS, have yet to be established. As a result, qualified Mohs Micrographic surgeons are few and far between; those who have, have received MMS training from another country. Right now, filling this void is not just a priority; it is an absolute necessity. Opportunities for training in MMS should be provided through specialized fellowships and training programs. These programs should be accredited by the CPSP and better if accredited by the Accreditation Council for Graduate Medical Education (ACGME), training dermatologists to acquire the necessary skills and knowledge for the application of specialized microsurgery techniques safely and effectively. Furthermore, dermatology residency programs should also place a greater emphasis on the incorporation of Mohs surgery training during the residency period, to ensure that the new dermatologists entering the field are well-versed. This is in accordance with the fact that one of many multiple cross-country surveys inquired dermatology residents regarding the choice of fellowship they would opt for. Approximately 70% replied in affirmation of dermatosurgery, which is still, unfortunately, non-existent [9]. Therefore, it is of the utmost importance that the faculty of dermatology in Pakistan introduces the subspecialty MMS FCPS in light of the dire need and to overcome the limitations in service delivery, as well as fulfill the dermatology trainees’ choice and demand to serve a rapidly growing population of more than 230 million people.

The demand for MMS is exceedingly on the rise as an increasing number of patients seek to undergo this procedure for the treatment of more complex and cosmetically challenging skin cancers. Hence, there is a need for additional dermatologists to be trained in MMS to fulfill the pressing requirement. By receiving adequate training in MMS, dermatologists would be able to provide their patients with the best possible care and have increased chances of successful outcomes. On top of that, they would have their reach extended to sophisticated lesions involving ever so cumbersome areas, which were previously and are largely still being dealt with by plastic surgeons to this date.
